# Epidemiological, Phenotypic, and Genomic Characterization of *Salmonella* from Food and Clinical Sources in Liaoning, China, 2022–2024

**DOI:** 10.3390/microorganisms14040823

**Published:** 2026-04-03

**Authors:** Mingyan Zhang, Lianzheng Yu, Menghan Li, Meimei Zhang, Weijie Wang, Haixia Liu, Yingzhi Geng, Miao Yu, Jinghong Ma, Qingyuan Wang, Wenli Diao, Yan Wang

**Affiliations:** 1 Liaoning Provincial Center for Disease Control and Prevention, Shenyang 110001, China; flyingbean@126.com (M.Z.);; 2 China National Center for Food Safety Risk Assessment, Beijing 100022, China

**Keywords:** *Salmonella*, antimicrobial resistance, genomic epidemiology, clinical sample, food sample

## Abstract

*Salmonella* is a major cause of foodborne illness worldwide, posing significant risks to public health and food safety. This study investigated the prevalence, serovar distribution, genotypic characteristics, and antimicrobial resistance (AMR) profiles of *Salmonella.* A total of 2515 food samples were collected from retail markets, supermarkets, and food processing facilities, and 13,670 stool samples were obtained from sentinel hospitals across 14 cities in Liaoning. The Kruskal–Wallis test was used to compare genetic features among serovars, followed by Dunn’s post hoc test for pairwise comparisons. A total of 314 *Salmonella* strains were identified, with raw poultry showing the highest detection rate (28.88%) among food sources and children aged 0–6 years (3.47%) the highest among the clinical age groups. Among food samples, *S.* Enteritidis was the most prevalent serovar (42.6%), and it was also the most common in clinical samples (35.8%); in contrast, *S.* 4,[5],12:i:- was dominant in pediatric clinical cases. According to AMR analysis, 90.13% of strains were resistant to at least one antibiotic and 67.83% were multidrug-resistant (MDR), with the highest resistance to ampicillin (68.47%). Analysis revealed that *S.* 4,[5],12:i:- harbored the ASSuT resistance module (blaTEM-1B, aph(3″)-Ib/aph(6)-Id, sul2, tet(B)). Extensive MDR phenotypes were observed in *S.* Indiana and *S.* Kentucky, associated with abundant insertion sequences (IS) and resistance genes (ARGs), including clinically critical determinants (blaNDM-9, mcr-1.1, rmtB). The highest mean virulence factor (VF) count (111.17) was observed in *S.* Enteritidis, contributing to its epidemiological success. Conversely, *S.* Indiana and *S.* Kentucky, predominantly food-associated serovars, exhibited reduced virulence but served as critical AMR reservoirs. These findings highlight the epidemiological characteristics and AMR risks of *Salmonella* in food and clinical settings, providing critical data for food safety and clinical antimicrobial stewardship.

## 1. Introduction

*Salmonella* remains a leading cause of foodborne illnesses worldwide, responsible for approximately 153 million annual gastroenteritis cases and over 60,000 deaths [1,2]. The genus encompasses over 2600 serovars [3], yet only about 100 account for most human infections [4]. Non-typhoidal *Salmonella* (NTS) causes manifestations ranging from self-limiting gastroenteritis to life-threatening invasive disease [5], with approximately 5% of patients (particularly infants, the elderly, and immunocompromised individuals) developing bacteremia or focal extra-intestinal infections [6]. Invasive complications (meningitis, osteomyelitis, endovascular infections) carry mortality rates up to 40–60% despite appropriate therapy [7]. Certain host-adapted serovars cause severe human disease and exhibit higher antimicrobial resistance (AMR), underscoring the need for serovar-level surveillance [8,9].

The acquisition of antimicrobial resistance (AMR) is driven by horizontal transfer of resistance determinants via mobile genetic elements, compounded by antibiotic overuse in medicine and animal husbandry [10]. Critically, AMR profiles vary substantially across serovars, necessitating serovar-specific surveillance. A nine-year national study across 26 Chinese provinces (2014–2022) identified *S.* Enteritidis (14.23%), *S.* Typhimurium (8.52%), *S.* Derby (6.30%), *S.* Kentucky (5.95%), and *S.* Rissen (5.24%) as predominant serovars in animal-origin foods, with marked regional distribution and dynamic prevalence shifts [11].

Of particular concern is escalating resistance to third-generation cephalosporins and fluoroquinolones—both WHO Highest Priority Critically Important Antimicrobials, with fluoroquinolones recommended as first-line therapy for invasive salmonellosis [12]. Genomic surveillance in Shanghai (2012–2021) revealed 56.6% of 458 clinical isolates exhibited multidrug resistance (MDR), with fluoroquinolone resistance mutations (*parC* T57S: 32.5%; *gyrA* D87Y: 29.0%) and *bla*_CTX-M-55_ (3.3%) widely disseminated [13]. In contrast, restrictive antibiotic policies in the USA and EU have maintained low resistance in livestock isolates (fluoroquinolones: 3.00% and 6.90%; third-generation cephalosporins: 4.20% and 0.40%) [14,15]. China, as the world’s largest antibiotic producer and consumer with over 50% consumption attributed to animal production [16], faces alarming resistance rates including MDR over 50% and clinically significant determinants such as *bla*_CTX-M-55_ across multiple regions [13,17,18].

Despite this global significance, comprehensive studies examining epidemiological distribution, serovar dynamics across diverse sources, and resistance profiles remain limited in northeastern China. Liaoning Province, a major agricultural region, represents a critical sentinel site for understanding *Salmonella* transmission at the human–animal–environment interface. Therefore, this study investigated (i) prevalence and seasonal distribution of *Salmonella* in food products and clinical diarrheal patients in Liaoning (2022–2024); (ii) serovar distribution patterns across sources and demographic groups; (iii) phenotypic and genotypic AMR profiles of predominant serovars; and (iv) associations between virulence factors, mobile genetic elements, and serovar-specific epidemiological success. The findings will inform evidence-based food safety policies and antimicrobial stewardship programs.

## 2. Materials and Methods

From January 2022 to December 2024, a total of 2515 food samples were collected from retail markets, supermarkets, and food processing facilities across Liaoning Province, China. Food samples were categorized into five groups: poultry (*n* = 419), pork (*n* = 75), beef (*n* = 207), aquatic products (*n* = 1200), all of which were raw, and other food sources including cooked meat products and raw sauces (*n* = 614). Poultry samples consisted of whole chicken carcasses, chicken breasts, or thighs, collected as retail cuts. Pork and beef samples included muscle tissue. Aquatic products primarily comprised fresh fish, shrimp and shellfish. All samples were transported to the laboratory in insulated containers with ice packs within 4 h and processed immediately upon arrival. In markets, food samples were stored under ambient or refrigerated conditions (4 °C) as per routine retail practice. Concurrently, 13,670 stool samples were collected from diarrheal patients with suspected foodborne illness at sentinel hospitals throughout Liaoning Province—covering all 14 of its cities. Four age groups were established for the clinical samples: 0–6 years (*n* = 2015), 6–18 years (*n* = 1235), 18–60 years (*n* = 7065), and >60 years (*n* = 3355).

*Salmonella* isolation from food samples was performed according to the National Food Safety Standard of China GB4789.4-2016 [19]. Briefly, 25 g of each food sample was pre-enriched in buffered peptone water at 37 °C for 18 h, followed by selective enrichment in tetrathionate broth and Rappaport Vassiliadis broth at 42 °C for 24 h. The enriched cultures were streaked onto xylose lysine deoxycholate agar and CHROMagar *Salmonella* plates, then incubated at 37 °C for 24 h. For clinical specimens, stool samples were directly inoculated onto *Salmonella Shigella* agar and MacConkey agar, followed by incubation at 37 °C for 24 h. Presumptive colonies were subsequently subjected to biochemical confirmation using the VITEK 2 system (bioMérieux, Marcy-l’Étoile, France). *Salmonella* serovars were determined by slide agglutination using commercial antisera (SSI Diagnostica, Hillerød, Denmark) according to the Kauffmann–White scheme. Serovar testing was performed by determining somatic O antigens and flagellar H antigens.

Phenotypic antimicrobial resistance profiles were determined by the broth microdilution method. The testing procedure was performed according to the Clinical and Laboratory Standards Institute (CLSI) M07 [20], and results were interpreted using CLSI M100 [21] breakpoints. A total of 20 antibiotics representing 12 antimicrobial classes were tested: Phenylpropanol class: CHL (Chloramphenicol); Quinolone class: NAL (Nalidixic acid) and CIP (Ciprofloxacin); Aminoglycoside class: GEN (Gentamicin) and AMK (Amikacin); Tetracycline class: TET (Tetracycline); Penicillin class: AMP (Ampicillin); Cephalosporins class: CFZ (Cefazolin), CXM (Cefuroxime), CPM (Cefepime), CAZ (Ceftazidime), CFX (Cefoxitin); Carbapenems class: IPM (Imipenem); β-lactam/β-lactam inhibitor complex:AMS (Ampicillin/Sulbactam), CTX/C (Cefotaxime/clavulanate); Macrolide class: AZM (Azithromycin); Folate pathway inhibitor class: SXT (Sulfamethoxazole/Trimethoprim); Polymyxin class: CT (Colistin), PB (Polymyxin). *Escherichia coli* ATCC 25922 was used as quality control strain. The minimal inhibitory concentration (MIC) was determined after incubation at 35 °C for 16–20 h. All phenotypic tests were performed in duplicate to ensure reproducibility, and results were recorded only when the quality control strain fell within acceptable MIC ranges. All antimicrobial agents were obtained from commercial sources (Shanghai Fuxing, Shanghai, China) and prepared according to CLSI guidelines. Multidrug resistance (MDR) was defined as resistance to three or more antimicrobial classes.

Genomic DNA was extracted from overnight bacterial cultures using the TIANamp Bacteria DNA Kit (Tiangen, Beijing, China) following the manufacturer’s instructions including enzymatic lysis with lysozyme (20 mg/mL) at 37 °C for 30 min, proteinase K digestion, and subsequent column-based purification. Genomic DNA quality and concentration were assessed by agarose gel electrophoresis and with a Qubit fluorometer (Thermo Fisher Scientific, Waltham, MA, USA). Whole-genome sequencing was performed on the Illumina xplus platform (PE 150). The average depth was 50× coverage.

Raw sequencing reads were trimmed for quality using Trimmomatic v0.39 and assembled using SPAdes v4.2.0. Genome quality was assessed with QUAST v5.3.0. Antimicrobial resistance genes (ARGs) were identified using ABRicate v1.0.1 against the ResFinder databases, with a minimum identity threshold of 80% and minimum coverage of 80%. Target gene mutations in quinolone resistance-determining regions (QRDRs) of *gyrA*, *gyrB*, *parC*, and *parE* were analyzed using PointFindertools (v3.0). Insertion sequence (IS) elements were identified using the ISfinder database through ABRicate. Virulence factor genes were detected using ABRicate against the Virulence Factor Database (VFDB) with the same thresholds. Multilocus sequence typing (MLST) was performed using MLST v2.19.0 based on the PubMLST scheme.

Statistical analyses were performed using R studio v4.1.2. The Kruskal–Wallis test was used to compare the mean numbers of antimicrobial resistance genes, insertion sequences, and virulence factors among serovars, followed by Dunn’s post hoc test for multiple comparisons. A *p*-value < 0.05 was considered statistically significant. Mean separation was indicated using superscript letters in tables. Figures were generated using itol online and R studio v4.1.2.

## 3. Results

### 3.1. The Detection Rate of Salmonella According to Different Classifications

From 2022 to 2024, a total of 2515 food samples were collected and classified into five categories based on isolated sources; 170 strains of *Salmonella* were detected from food samples (Table 1). Poultry exhibited the highest *Salmonella* detection rate at 28.88% (121/419). Pork had a detection rate of 22.67% (17/75), followed by beef 4.83% (10/207). Other food sources (cooked meat products, raw sauce) had a detection rate of 1.63% (10/614); 144 strains of *Salmonella* were detected from 13670 diarrheal patients with suspected foodborne illness. Children aged 0–6 years had the highest *Salmonella* detection rate among clinical cases at 3.47% (70/2015).

*Salmonella* detection rates in food samples showed a distinct seasonal pattern (Table 2), with the highest rate observed in summer, at 14.06% (89/633), followed by autumn, at 6.64% (42/633), spring, at 4.01% (25/624), and winter, at 2.24% (14/625). In clinical samples, summer had the highest number of positive clinical samples (91 cases), exceeding spring (22), autumn (29), and winter (2). During summer, there is an increase in the number of diarrheal patients with suspected foodborne illness. The detection rate in summer (1.24%) was lower than spring (2.02%) due to a 6.7-fold larger sample size (7346 vs. 1090), but the absolute burden of clinical cases remained highest in summer. In both the clinical and food samples, summer appears to be a season of significance. This suggests that environmental factors in summer, such as higher temperatures and humidity, may contribute to the proliferation and spread of *Salmonella* in both clinical and food-related contexts.

### 3.2. The Distribution of Salmonella Serovars with Different Classifications

Among food sources, different serovars show distinct prevalence patterns (Table 1). In poultry, *S.* Enteritidis is the most dominant serovar, accounting for 53.72% of the detected *Salmonella* isolates, followed by *S.* Kentucky (10.74%). For pork, *S.* Infantis and *S.* London are the most common, each making up 23.53% of the positive samples. The “other food sources” category includes cooked meat products and raw sauces, in which *S*. 4,[5],12:i:- showed a relatively higher prevalence (40.00%), suggesting potential post-processing contamination. *Salmonella* Enteritidis accounted for 20.00% in this category.

**Figure 1:**
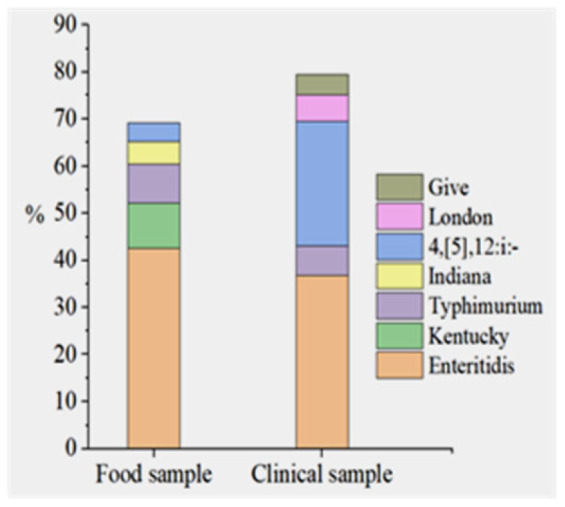
Prevalence distribution of top five *Salmonella* serovars in food and clinical samples.

**Figure 2:**
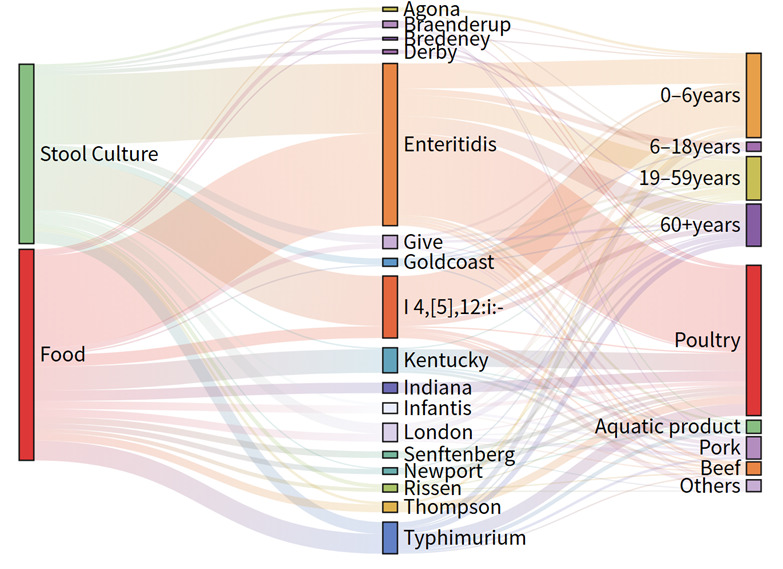
Distribution patterns of *Salmonella* serovars in different classifications.

In the 0–6-year age group from clinics, *S.* 4,[5],12:i:—is the most common serovar, representing 41.43% of the detected isolates, while *S.* Enteritidis is the second most prevalent at 27.14%. Across most categories, *S.* Enteritidis was the dominant serovar, except for the 0–6 age group, where *S.* 4,[5],12:i:- prevailed. The top five serovars from both sources are presented in Figure 1. In both food and clinical samples, *S.* Enteritidis was the most prevalent serovar, accounting for 42.6% and 35.8%, respectively. Additionally, *S.* Typhimurium and *S.* 4,[5],12:i:- were detected in the top five serovars from both sources. The Sankey plot (Figure 2) and Table 1 shows that the distribution of *Salmonella* serovars is uneven among different sample types (food and stool cultures), age groups, and food categories. With a total of 126 isolates, *S.* Enteritidis shows a balanced distribution between food (57.94%) and stool samples (42.06%), indicating its role in both food contamination and clinical cases. Among the serovars, *S.* Indiana was exclusively isolated from food sources (100%), whereas *S.* Kentucky, while also predominantly food-associated (94.12%), was the only one of the two detected in clinical samples, albeit at a very low frequency (5.88%). The highest proportion in stool samples (84.44%) was observed for *S.* 4,[5],12:i:-, with only 15.56% from food, highlighting its role in human infections.

### 3.3. Antibiotic Susceptibility of Salmonella Isolates

**Figure 3:**
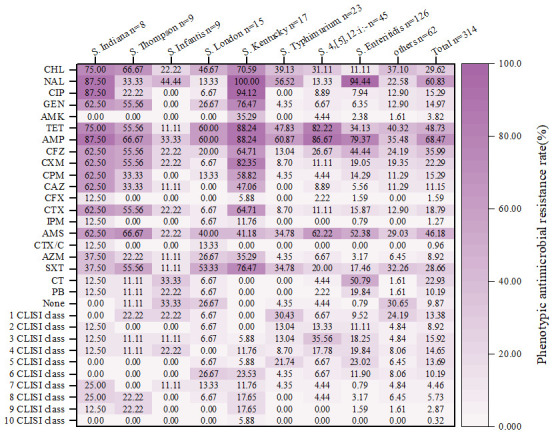
The phenotypic antimicrobial resistance rate of the predominant serovars.

**Figure A1:**
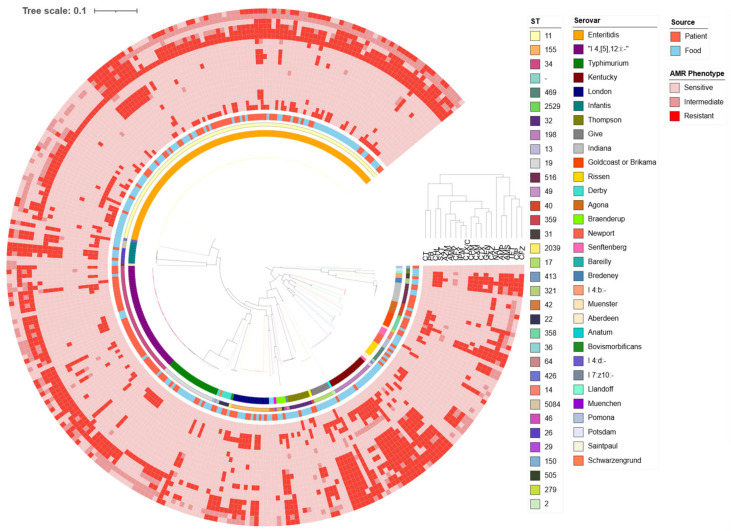
This circular phylogenetic tree illustrates the evolutionary relationships among different serovars of *Salmonella*, presenting a multi-layered view of relevant characteristics. The innermost ring is color-coded to represent different *Salmonella* serovars. The second ring from the inside showcases the ST of the *Salmonella* isolates. The third ring from the inside indicates the source of the *Salmonella* isolates. The outermost ring displays the antimicrobial resistance (AMR) phenotype. From inner to outer, the drugs corresponding to the resistance markers are as follows: CT, PB, CHL, SXT, AZM, AMK, CFX, IPM, CTX/C, CPM, CXM, CTX, GEN, CAZ, NAL, AMP, AMS, TET, CIP, CFZ.

The AMR phenotype and AMR rate are presented in Figure A1 and Figure 3. Twenty antibiotics have been classified into twelve categories according to the 2024 version of the Clinical and Laboratory Standards Institute (CLSI) guidelines, Edition 34. Among a total of 314 *Salmonella* strains, the proportion of strains resistant to at least one antibiotic is 90.13%. The proportion of multidrug-resistant (MDR) strains, which are resistant to more than three CLSI class of antibiotics, is 67.83%. All *Salmonella* strains exhibit the highest resistance rate to AMP, which is 68.47%, followed by NAL (60.83%), TET (48.73%), and AMS (46.18%).

Some serovars show relatively high resistance rates to certain antimicrobials. Relatively high resistance rates to NAL were observed for *S.* Kentucky, *S.* Enteritidis, and *S.* Indiana, with the rates being 100%, 94.44%, and 87.50%, respectively. For another quinolone class antibiotic, CIP, the resistance rates of *S.* Kentucky and *S.* Indiana are 94.12% and 87.50%, respectively, which are significantly higher than those of other serovars. For TET, the resistance rates of *S.* Kentucky, *S.* 4,[5],12:i:, and *S.* Indiana are 88.24%, 82.22%, and 75%, respectively. Among the drugs tested, AMP has the highest resistance rate, with the resistance rates of *S.* Kentucky, *S.* 4,[5],12:i:, *S.* Indiana, and *S.* Enteritidis to AMP all exceeding 75%. Relatively high resistance rates to CAZ and CTX (third-generation cephalosporins: ceftazidime and cefotaxime) were also displayed by *S.* Indiana, *S.* Kentucky, and *S.* Thompson, which warrants vigilance. Most serovars are sensitive to amikacin (AMK), whereas the resistance rate of *S.* Kentucky to AMK is as high as 35.29%. Among the 12 CLSI classes of antibiotics, the highest level of resistance detected was to 10, observed in one strain of *S.* Kentucky isolated from poultry. Next, resistance to nine CLSI classes of antimicrobials was noted, with *S.* Indiana, *S.* Kentucky, and *S.* Thompson each accounting for more than 10% of such resistant isolates. For MDR (resistance to over three CLSI classes of antimicrobials), the highest resistance rates were seen in *S.* Kentucky (100%), followed by *S.* Indiana (87.5%), *S.* Enteritidis (78.57%), *S.* 4,[5],12:i:—(75.56%), *S.* Thompson (66.67%), *S.* London (60%), *S.* Typhimurium (52.17%), *S.* Infantis (44.44%), and others (40.32%).

### 3.4. Genotypic AMR Profiles of Predominant Salmonella Serovars

A total of 66 AMR genes were identified among 314 *Salmonella* isolates, conferring resistance to 10 different antimicrobial classes (Table A1). The distribution of these genes was highly heterogeneous, with distinct serovar-specific patterns and varying prevalence rates.

Aminoglycoside resistance was widespread across all serovars, with *aac(6′)-Iaa* detected in 100% of isolates across all tested serovars, indicating a conserved resistance mechanism. However, *S.* Indiana exhibited the highest prevalence of aminoglycoside resistance genes, including *aac(3)-IVa* (75.00%), *aph(4)-Ia* (75.00%), and *ARR-3* (75.00%). High carriage of *aac(3)-IId* (64.71%), *aadA7* (88.24%), and *ARR-3* (64.71%) was observed in *S.* Kentucky, while *S.* 4,5,12:i:- and *S.* Thompson had a notable prevalence of *aph(3*″*)-Ib* (91.11% and 66.67%, respectively) and *aph(6)-Id* (91.11% and 66.67%, respectively).

β-lactam resistance was dominated by *bla*_TEM-1B_, which was highly prevalent in *S.* Enteritidis (73.02%), *S.* Typhimurium (52.17%), *S.* 4,5,12:i:- (77.78%), and *S.* London (60.00%). The *bla*_CTX-M-55_ was the most broadly distributed ESBL gene, found in *S.* Indiana (50.00%), *S.* Kentucky (52.94%), *S.* Thompson (33.33%), and others. *S.* Kentucky uniquely harboring *bla*_CTX-M-14b_ (23.53%) was observed, while *S.* Thompson and *S.* Infantis showed a preference for *bla*_CTX-M-65_ (44.44% and 33.33%, respectively). The plasmid-mediated AmpC gene *bla*_CMY-2_ was only detected in *S.* London (6.67%). Of particular clinical concern, *bla*_NDM-9_, a carbapenemase gene conferring resistance to last-line antibiotics, was detected exclusively in *S.* Indiana (12.50%).

Sulfonamide resistance was common, with *sul2* detected in *S.* Enteritidis (47.62%), *S.* Typhimurium (34.78%), *S.* 4,5,12:i:- (86.67%), and *S.* Indiana (87.50%). *sul1* was particularly prevalent in *S.* Indiana (75.00%) and *S.* Kentucky (94.12%), suggesting co-selection with other resistance determinants.

Tetracycline resistance was primarily mediated by *tet(A)*, which was highly prevalent in *S.* Indiana (87.50%), *S.* Kentucky (88.24%), and *S.* London (73.33%). A unique pattern was observed in *S.* 4,5,12:i:-, where *tet(B)* was detected in 80.00% of isolates, indicating a distinct resistance mechanism.

Plasmid-mediated quinolone resistance (PMQR) genes were prevalent. *qnrS1* was the most common, detected in nearly all serovars, with the highest rates in *S.* Thompson (77.78%), followed by *S.* London (40.00%) and *S.* Typhimurium (34.78%). The bifunctional gene *aac(6′)-Ib-cr*, which confers resistance to both aminoglycosides and ciprofloxacin, was found at alarmingly high rates in *S.* Indiana (75.00%), *S.* London (40.00%), and *S.* 4,[5],12:i:- (13.33%). The *oqxAB* efflux pump genes were primarily confined to *S.* Indiana (37.50%). Otherwise, the bacterial genes *gyrA, gyrB, parC*, and *parE* encode subunits of two essential type II topoisomerases: DNA gyrase (*gyrA* and *gyrB*) and topoisomerase IV (*parC* and *parE*). Point mutations within these genes, particularly in conserved regions designated as the quinolone resistance-determining regions (QRDRs), constitute a principal mechanism of acquired antimicrobial resistance. Mutations in the *gyrB* and *parE* gene were not detected across 314 isolations (Table A2). Remarkable variability in *gyrA* and *parC* mutation rates was observed among the serovars. Among the serovars, *S.* Indiana, *S.* Kentucky and *S.* Infantis also exhibited high mutation burdens, with *S.* Indiana showing 100% *gyrA* and 87.5% *parC* mutations, *S.* Kentucky showing 100% *gyrA* and 94.12% *parC*, and *S.* Infantis showing 100% *parC* and 33.33% *gyrA mutations*. A very high *gyrA* mutation rate (94.44%) but a very low *parC* mutation rate (5.56%) was displayed by *S.* Enteritidis, indicating that single *gyrA* mutations may be the primary mechanism for quinolone resistance in this serovar. With minimal mutations in *gyrA* (4.44%) and *parC* (4.44%), *S.* 4,[5],12:i:- exhibited the lowest overall mutation burden.

Regarding macrolide resistance genes, *S.* Indiana exhibited the highest prevalence, with 75.00% of isolates carrying *mph(A)*. Moreover, this serovar was the only one in which *mph(E)* and *msr(E)* were detected (both at 12.50%). Notable levels of macrolide resistance were also shown by *S.* Kentucky and *S.* London, primarily driven by the mph(A) gene, with prevalence rates of 41.18% and 40.00%, respectively.

Strikingly, *S.* Indiana exhibited the highest prevalence of *mcr-1.1*, with 50.00% of its isolates carrying this gene. Simultaneously, it was the exclusive carrier of *bla*_NDM-9_ (12.50%), further cementing its status as a critical AMR reservoir.

### 3.5. Genotypic Features Among Different Salmonella Serovars

**Figure 4:**
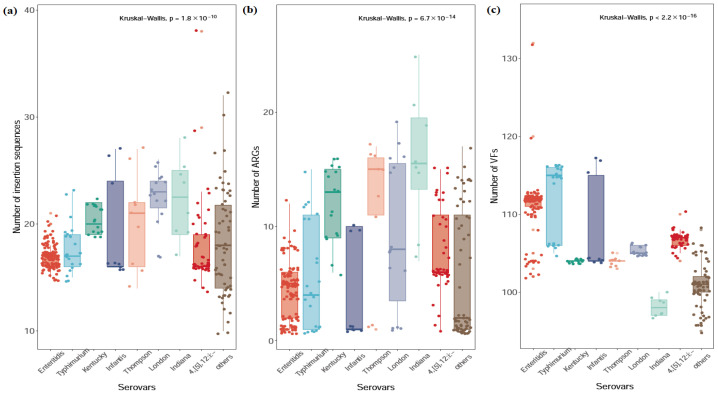
The comparation of genotypic features among different predominant *Salmonella* serovars in (**a**) Number of insertion sequences; (**b**) Number of ARGs; (**c**) Number of VFs.

The mean number of ARGs varied significantly among the serovars, ranging from 4.00 to 15.75 (Figure 4 and Table A3). S. Indiana exhibited the highest mean ARG count 15.75,n=8, significantly exceeding all other serovars (*p* < 0.05), indicating a markedly enriched resistome, followed by *S.* Kentucky (11.94, *n* = 17), *S.* Thompson (11.67, *n* = 9) and *S.* London (9.20, *n* = 15). In contrast, *S.* Enteritidis (4.30, *n* = 126), S. Infantis (4.00, *n* = 9), and other serovars (5.44, *n* = 62) harbored the lowest ARG counts, suggesting a more limited intrinsic resistance repertoire. *Salmonella* Typhimurium (5.70, *n* = 23) and *S.* 4,[5],12:i:- (7.73, *n* = 45) showed intermediate ARG levels.

A positive association was observed between high IS element counts and elevated ARG numbers. *S.* Indiana again demonstrated the highest mean IS element count (22.25), supporting a potential role of IS-driven genomic plasticity in ARG acquisition. Elevated IS counts were also observed in *S.* London (22.33), *S.* Kentucky (20.47), *S.* Thompson (20.44), and *S*. Infantis (19.22), suggesting enhanced mobilome activity in these serovars. Lower IS loads were found in *S.* Enteritidis (16.91), S. Typhimurium (17.83), *S.* 4,[5],12:i:- (18.13), and other serovars (18.18), indicating relatively stable genomic structures.

The Kruskal–Wallis test revealed a statistically significant difference in the mean number of virulence factors (VFs) across the *Salmonella* serovars (*p* < 2.2 × 10^−16^). As detailed in Table A3, *S.* Enteritidis and *S.* Typhimurium harbored the highest mean number of VFs, with 111.17 and 112.13, respectively. These were followed closely by *S.* Infantis (108.11) and *S.* 4,[5],12:i:- (107.06). Serovars such as *S.* Thompson, *S.* London, and other minor serovars exhibited moderate mean VF counts, ranging from 100.77 to 105.33. *S.* Indiana and *S.* Kentucky displayed the lowest mean number of VFs among the analyzed serovars, with a mean of 98.13.

## 4. Discussion

In this study, 314 strains of *Salmonella* isolated from food and clinical sources exhibited eight predominant serovars in Liaoning China between 2022 and 2024. *Salmonella* Enteritidis, the most prevalent serovar overall, exhibited broad epidemiological relevance, being detected across multiple food sources at 42.94% (73/170) and all clinical age groups at 35.81%(53/144). It was also the predominant serovar in raw poultry in this study, accounting for 53.72% of positive samples. This contrasts sharply with the serovar distribution in U.S. poultry, where *S.* Kentucky is dominant (35.47%) and *S.* Enteritidis ranks only third [22]. The predominance of *S.* Enteritidis in clinical isolates (35.81%) was similarly higher than in U.S. data (27.81%), mirroring the pattern observed in poultry [23].

*Salmonella* 4,[5],12:i:- is a monophasic variant of *S.* Typhimurium. Since the serotype first caused a major outbreak in Spain in the mid-1990s, it has rapidly spread globally [24]. Over the past two decades, it has become one of the top five serotypes responsible for human and animal infections [25]. Unlike other serovars prevalent in raw meat, this serovar was rarely detected there but showed a strong link to cooked meat and clinical cases, particularly in vulnerable populations (41.43% of cases in ages 0–6 years; 15.15% in >60 years), suggesting that contamination occurs later in the food chain. Accounting for 59% of cases in the United States and 28% in the United Kingdom, it has emerged as a dominant cause of human salmonellosis globally, consistent with its substantial burden in both the United States and Europe [26].

In our study, nearly all *S.* Indiana (100%) and *S.* Kentucky (94.12%) isolates originated from poultry with only rare clinical detection (Table A4, Figure 2), underscoring their strong food source predominance. A 2021–2024 duck study reported consistent findings, identifying *S.* Indiana (21.93%) and *S.* Kentucky (18.42%) as predominant serovars in poultry, with most isolates exhibiting high resistance to multiple antibiotics [27]. Consistent with their rare clinical detection in this study, all *S.* Kentucky isolates belonged to the poultry-adapted strain (ST198, n=17), which only occasionally infects humans [28,29].

The pronounced seasonal variation, with prevalence peaking in summer for both food (14.06%) and clinical samples (91 cases), reflects the environmental influence on *Salmonella*. Higher ambient temperatures during summer promote bacterial replication in food matrices and increase human exposure through outdoor food preparation and consumption patterns [30,31]. Our observation reflects the true burden of diarrheal illness during this season.

The overall prevalence of antimicrobial resistance in this study (90.13% AMR, 67.83% MDR) reflects the continued selective pressure exerted by antibiotic use. This pressure is especially pronounced in China, which ranks as the world’s largest producer and consumer of antibiotics, where the share used in animals accounts for more than half of the total consumption [32].

Aminoglycoside resistance genes were the most prevalent and diverse category identified in foodborne *Salmonella* isolates across multiple continents [33]. The gene *aac(6′)-Iaa* was universally present (100%) in all serovars. *S.* Kentucky (ST198) harbored the most diverse genes, with high carriage rates of *aac(3)-Id* (17.65%), *aac(3)-IId* (64.71%), *aadA17* (52.94%), *aadA7* (88.24%), and *rmtB* (17.65%), a 16S rRNA methylase conferring high-level pan-aminoglycoside resistance. The presence of *rmtB* is particularly concerning due to its potential for horizontal transfer and its association with clinically significant resistance. Recent genomic surveillance from China has similarly documented *aac(3)-Id*, *aadA2*, *aadA7*, and *rmtB* in *S.* Kentucky (ST198) isolates from both human clinical cases and food sources, confirming the persistence of this resistance profile in China [34,35].

The β-lactamase gene profile was dominated by the broad-spectrum *bla*_TEM-1B_, which was present in the majority of serovars, particularly *S.* Enteritidis (73.02%), *S.* 4,[5],12:i:- (77.78%), and *S.* London (60.00%). The World Health Organization has classified ESBL-producing Enterobacteriaceae as high-priority pathogens, underscoring the global significance of these findings. In this study, *bla*_CTX-M-55_, a common ESBL variant in food animals, was detected in *S.* Indiana (50.00%), *S.* Kentucky (52.94%), and *S.* Thompson (33.33%). The detection of *bla*_CTX-M-14_, *bla*_CTX-M-65_, and *bla*_CTX-M-123_ highlights the diversity of ESBL genes circulating in these serovars. One *S.* Indiana strain (ST17) isolated from chicken carried *bla*_NDM-9_, a carbapenemase gene conferring resistance to last-line carbapenem antibiotics. This finding aligns with the recent and ongoing emergence of extensively drug-resistant *S.* Indiana harboring *bla*_NDM-9_ across multiple regions in China, including Fujian and Shanghai, raising concerns for public health [36,37,38].

Resistance to folate pathway inhibitors was mediated by a combination of *sul* and *dfrA* genes. *sul1* and *sul2* were the predominant sulfonamide resistance genes. The *sul2* gene was particularly frequent in *S.* 4,[5],12:i:- (86.67%) and *S.* Indiana (87.50%), while *sul1* was ubiquitous in *S.* Kentucky (94.12%). The *dfrA* genes (conferring trimethoprim resistance) showed serovar-specific associations. The *dfrA14* gene was common in *S.* Kentucky (64.71%) and *S.* Thompson (66.67%), while *dfrA12* was associated with *S.* Typhimurium (21.74%) and *S.* London (33.33%).

Tetracycline resistance was primarily conferred by *tet(A)* and *tet(B)*. The *tet(A)* gene was widespread, with extremely high prevalence in *S.* Indiana (87.50%), *S.* Kentucky (88.24%), and *S.* London (73.33%). In contrast, *tet(B)* was almost exclusively found in *S.* 4,[5],12:i:- (80.00%). This pronounced serovar-specific partitioning is consistent with global observations, wherein *S.* 4,[5],12:i:- frequently harbors *tet(B)* as an integral component of the classical ASSuT MDR module [26,39]. In addition to tet(B), this module comprises *bla*_TEM-1B_ (77.78%), *aph(3*″*)-Ib/aph(6)-Id* (91.11%), and *sul2* (86.67%) in this study, collectively conferring resistance to ampicillin, streptomycin, sulfisoxazole, and tetracycline.

Beyond the stable core ASSuT module, our data suggest that this successful clone is increasingly acting as a platform for the acquisition of additional resistance determinants. Within the *S.* 4,[5],12:i:-, we observed notable co-carriage of *ARR-3* (24.44%), which confers rifampicin resistance, as well as the plasmid-mediated quinolone resistance (PMQR) genes *aac(6′)-Ib-cr* (13.33%) and *qnrS1* (26.67%). This evolutionary trajectory mirrors findings from international surveillance; for instance, a U.S. study reported that 16% of isolates within the MDR clade harbored genetic determinants associated with decreased susceptibility to ciprofloxacin, ceftriaxone, or azithromycin [40].

Regarding macrolide resistance, *S.* Indiana exhibited the highest carriage rate of mph(A) at 75.00%, followed by *S.* Kentucky (41.18%) and *S.* London (40.00%). In contrast to *S.* Indiana and *S.* Kentucky, which were predominantly poultry-associated, *S.* London in this study was primarily recovered from pork samples (Table 1). A recent surveillance of retail pork in Sichuan, China, revealed *S.* London as the dominant serovar, exhibiting a 47.83% carriage rate of the *mph(A)* gene [41]. The *mph(A)* carriage rates in China far exceed azithromycin resistance prevalence in the EU (1.90%) and US (4.09%) [15,16], highlighting stronger selective pressure for macrolide resistance in Chinese swine and poultry sectors.

PMQR genes and target mutations were widely distributed. The *qnrS1* gene was prevalent, peaking in *S.* Thompson (77.78%) and *S.* London (40.00%). The *aac(6′)-Ib-cr* gene was alarmingly high in *S.* Indiana (75.00%), *S.* London (40.00%), and *S.* 4,[5],12:i:- (13.33%), while *oqxAB* was mainly confined to *S.* Indiana (37.50%). The *gyrA* gene mutations correlated strongly with NAL resistance: *S.* Indiana and *S.* Kentucky exhibited 100% *gyrA* mutations and complete NAL resistance, followed by *S.* Enteritidis (94.44% for both). Consistent with previous reports [42,43], *gyrA* mutations alone did not confer CIP resistance without *parC* alterations. Accordingly, *S.* Indiana, *S.* Kentucky, and *S.* Thompson exhibited nearly complete CIP resistance (95–100%) alongside high *parC* mutation rates, underscoring the critical role of *parC* in fluoroquinolone resistance.

Quantitative analysis revealed fundamental, serovar-specific differences in resistance architecture: AMR distribution correlates with distinct mobile genetic element burdens and gene repertoires [44]. This association was evident in the highest-burden serovars: *S.* London (22.33 IS; 9.20 ARGs), *S.* Indiana (22.25; 15.75), and *S.* Kentucky (20.47; 11.94). Conversely, *S.* Enteritidis displayed the lowest mean IS element count (16.91) and the second-lowest ARG burden (4.30). The MDR rates across serovars in our study revealed distinct groupings.

Our analysis of the mean number of VFs across different serovars (Table A3 and Figure 4) revealed a high degree of concordance with their prevalence in clinical isolates. *S.* Enteritidis, the most frequently isolated serovar from clinical samples (37.5%), possessed the highest mean VF count among all serovars analyzed (111.17). This finding aligns with previous genomic studies demonstrating that *S.* Enteritidis consistently harbors a complete set of pathogenicity islands (SPIs 1–5, 12–14, 16–17) and plasmid-encoded virulence genes that contribute to its epidemiological success [45]. *Salmonella* 4,[5],12:i:-, ranking second in clinical samples (27.08%), demonstrated a mean VF count of 107.06. Conversely, serovars such as *S.* Kentucky and *S.* Indiana, which were prominent in food samples, but not among the top clinical isolates, exhibited the lowest mean VF counts (104.00 and 98.13, respectively). This inverse relationship between food prevalence and VF counts suggests that while these serovars successfully contaminate the food supply, their reduced virulence potential limits their ability to cause human disease [46]. However, the presence of elevated antimicrobial resistance gene (ARG) and insertion sequence (IS) loads in *S.* Kentucky and *S.* Indiana—despite their lower VF counts—underscores their importance in risk assessment and surveillance. These genetic features indicate adaptation to agricultural environments where antimicrobial selection pressure is intense, and they retain the potential to serve as reservoirs for resistance determinants that could be horizontally transferred to more virulent serovars [47]. Thus, the convergence of resistance determinants in these serovars poses a persistent threat, warranting targeted interventions beyond virulence-based risk assessment. To mitigate the spread of Salmonella and its antimicrobial resistance, targeted interventions should include enhanced surveillance during peak summer months and prudent antibiotic use in livestock production, particularly in poultry farming. These measures are essential to reduce the burden of multidrug-resistant serovars such as *S.* Indiana and *S.* Kentucky.

## 5. Conclusions

This comprehensive genomic surveillance conducted in Liaoning Province from 2022 to 2024 elucidates the intricate epidemiological and resistome profiles of non-typhoidal Salmonella (NTS) at the human–food interface. Our findings demonstrate a pronounced seasonal peak in summer, underscoring the role of environmental factors in Salmonella transmission. The serovar distribution was markedly source-specific: while *S.* Enteritidis was the predominant serovar across both food and clinical settings, *Salmonella* 4,[5],12:i:- exhibited a unique epidemiological niche, rarely detected in raw meat but predominantly isolated from cooked/processed food products and clinical samples from vulnerable populations, and genomically harbored the classical ASSuT resistance module. Alarmingly, the study reveals a high prevalence of multidrug resistance (67.83%), with specific serovars like *S.* Indiana and *S.* Kentucky serving as critical reservoirs of clinically significant resistance genes, including *bla*_NDM-9_, *mcr-1.1*, and *rmtB*. These serovars exhibited extensive MDR phenotypes linked to high ARGs and IS loads, indicating their role as platforms for horizontal gene transfer. Conversely, the epidemiological success of *S.* Enteritidis appears to be driven by its high virulence factor (VF) load rather than extensive AMR.

These findings underscore the urgent need for integrated “One Health” surveillance bridging food safety and clinical antimicrobial stewardship. Targeted interventions in livestock production, particularly poultry farming, are critical to mitigating the public health threat posed by multidrug-resistant *Salmonella*. An emphasis should be placed on seasonal risk communication, post-processing food hygiene, and antimicrobial stewardship programs to address the distinct transmission dynamics and resistance reservoirs identified in this study.

## Figures and Tables

**Table 1:** The detection rate and prevalent serovars under the different classifications.

Classification	Subcategory	Number of Positive Samples	Total Samples Tested	Detection Rate (%)	Most Prevalent Serovar (1st)	Second Most Prevalent Serovar (2nd)
Source (food)	Poultry	121	419	28.88	*S.* Enteritidis (53.72%)	*S.* Kentucky (10.74%)
	Pork	17	75	22.67	*S.* London (23.53%)	*S.* Infantis (23.53%)
	Beef	10	207	4.83	*S.* Kentucky (20.00%)	*S.* Typhimurium (20.00%)
	Aquatic products	12	1200	1.00	*S.* Typhimurium (25.00%)	*S.* Enteritidis (16.67%)
	Others	10	614	1.63	*S.* 4,[5],12:i:- (40.00%)	*S.* Enteritidis (20.00%)
Age group (clinics)	0–6 years	70	2015	3.47	*S.* 4,[5],12:i:- (41.43%)	*S.* Enteritidis (27.14%)
	6–18 years	7	1235	0.57	*S.* Enteritidis (71.43%)	*S.* Goldcoast (14.29%)
	18–60 years	33	7065	0.47	*S.* Enteritidis (48.48%)	*S.* 4,[5],12:i:- (15.15%)
	>60 years	34	3355	1.01	*S.* Enteritidis (38.24%)	*S.* 4,[5],12:i:- (11.76%)

**Table 2:** Seasonal Distribution of *Salmonella* Detection Rates in Food and Clinical Samples.

Sample Type	Season	Number of Positive Samples	Total Samples Tested	Detection Rate (%)
Food sample	Spring	25	624	4.01
	Summer	89	633	14.06
	Autumn	42	633	6.64
	Winter	14	625	2.24
Clinical sample	Spring	22	1090	2.02
	Summer	91	7346	1.24
	Autumn	29	4476	0.65
	Winter	2	758	0.26

## Data Availability

The original contributions presented in this study are included in the article. Further inquiries can be directed to the corresponding authors.

## Appendix A

**Table A1:** The genotypic AMR profiles per isolate of the predominant *Salmonella* serovars.

Classifications	Genes	Genotypic AMR Rate (%)								
		*S.* Enteritidis (*n* = 126)	*S.* Typhimurium (*n* = 23)	*S.* 4,[5],12:i:- (*n* = 45)	*S.* Indiana (*n* = 8)	*S.* Infantis (*n* = 9)	*S.* Kentucky (*n* = 17)	*S.* London (*n* = 15)	*S.* Thompson (*n* = 9)	Others (*n* = 62)
Aminoglycosides	*aac(3)-Id*	0.00	0.00	0.00	0.00	0.00	17.65	0.00	0.00	0.00
	*aac(3)-IIa*	0.00	0.00	0.00	0.00	0.00	0.00	0.00	0.00	1.61
	*aac(3)-IVa*	0.00	0.00	6.67	75.00	33.33	0.00	6.67	44.44	6.45
	*aac(3)-IId*	2.38	0.00	4.44	0.00	0.00	64.71	40.00	22.22	8.06
	*aac(6′)-Iaa*	100.00	100.00	100.00	100.00	100.00	100.00	100.00	100.00	100.00
	*aadA1*	0.00	0.00	0.00	0.00	0.00	0.00	0.00	0.00	4.84
	*aadA16*	0.00	8.70	11.11	0.00	0.00	0.00	40.00	0.00	3.23
	*aadA17*	0.00	0.00	0.00	0.00	0.00	52.94	0.00	0.00	0.00
	*aadA2*	0.00	21.74	0.00	12.50	0.00	0.00	0.00	44.44	19.35
	*aadA22*	0.00	0.00	0.00	0.00	0.00	0.00	0.00	0.00	1.61
	*aadA5*	10.32	0.00	0.00	37.50	0.00	0.00	0.00	0.00	1.61
	*aadA7*	0.00	0.00	0.00	0.00	0.00	88.24	0.00	0.00	0.00
	*aadA8b*	0.00	0.00	0.00	0.00	0.00	0.00	33.33	0.00	0.00
	*ant(3″)-Ia*	1.59	21.74	6.67	12.50	33.33	17.65	6.67	33.33	17.74
	*aph(3′)-Ia*	0.00	0.00	4.44	37.50	33.33	100.00	0.00	66.67	14.52
	*aph(3″)-Ib*	58.73	26.09	91.11	37.50	0.00	17.65	40.00	44.44	4.84
	*aph(3′)-IIa*	3.97	0.00	0.00	0.00	0.00	0.00	0.00	0.00	0.00
	*aph(4)-Ia*	0.00	0.00	6.67	75.00	33.33	0.00	0.00	44.44	6.45
	*aph(6)-Id*	58.73	26.09	91.11	62.50	0.00	17.65	40.00	66.67	16.13
	*rmtB*	0.00	0.00	0.00	0.00	0.00	17.65	0.00	0.00	0.00
	*armA*	0.00	0.00	0.00	12.50	0.00	0.00	0.00	0.00	0.00
	*ARR-3*	0.00	8.70	24.44	75.00	0.00	64.71	33.33	66.67	20.97
β-lactams	*bla*_CMY-2_	0.00	0.00	0.00	0.00	0.00	0.00	6.67	0.00	0.00
	*bla*_CTX-M-123_	0.00	0.00	0.00	12.50	0.00	0.00	0.00	0.00	0.00
	*bla*_CTX-M-14_	7.94	0.00	4.44	0.00	0.00	0.00	0.00	0.00	0.00
	*bla*_CTX-M-14b_	0.00	0.00	0.00	0.00	0.00	23.53	0.00	0.00	0.00
	*bla*_CTX-M-27_	0.00	4.35	0.00	0.00	0.00	0.00	0.00	0.00	0.00
	*bla*_CTX-M-55_	3.17	0.00	2.22	50.00	0.00	52.94	0.00	33.33	9.68
	*bla*_CTX-M-64_	0.00	4.35	0.00	0.00	0.00	0.00	0.00	0.00	0.00
	*bla*_CTX-M-65_	0.00	0.00	8.89	25.00	33.33	0.00	0.00	44.44	3.23
	*bla*_NDM-9_	0.00	0.00	0.00	12.50	0.00	0.00	0.00	0.00	0.00
	*bla*_OXA-1_	0.00	0.00	2.22	75.00	0.00	0.00	0.00	0.00	1.61
	*bla*_OXA-10_	0.00	0.00	11.11	0.00	0.00	0.00	6.67	44.44	4.84
	*bla*_TEM-127_	0.79	0.00	0.00	0.00	0.00	0.00	0.00	0.00	0.00
	*bla*_TEM-141_	3.17	0.00	0.00	0.00	0.00	41.18	0.00	0.00	0.00
	*bla*_TEM-1A_	0.00	0.00	0.00	0.00	0.00	0.00	0.00	0.00	1.61
	*bla*_TEM-1B_	73.02	52.17	77.78	25.00	0.00	11.76	60.00	33.33	24.19
Phenylpropanols	*catA2*	0.00	0.00	0.00	0.00	0.00	0.00	20.00	0.00	0.00
	*catB3*	0.00	0.00	2.22	75.00	0.00	0.00	0.00	0.00	1.61
	*cmlA1*	0.79	21.74	11.11	0.00	0.00	0.00	6.67	33.33	19.35
	*floR*	2.38	34.78	35.56	62.50	33.33	76.47	46.67	66.67	32.26
Sulfonamides	*dfrA1*	0.00	0.00	0.00	0.00	0.00	5.88	0.00	0.00	0.00
	*dfrA12*	0.00	21.74	0.00	25.00	0.00	0.00	33.33	0.00	14.52
	*dfrA14*	1.59	0.00	15.56	0.00	33.33	64.71	6.67	66.67	17.74
	*dfrA17*	10.32	0.00	0.00	37.50	0.00	0.00	0.00	0.00	1.61
	*dfrA27*	0.00	8.70	11.11	0.00	0.00	0.00	40.00	0.00	3.23
	*sul1*	0.00	8.70	11.11	75.00	33.33	94.12	40.00	0.00	8.06
	*sul2*	47.62	34.78	86.67	87.50	0.00	0.00	40.00	0.00	20.97
	*sul3*	0.00	21.74	0.00	12.50	0.00	0.00	33.33	66.67	24.19
Fosfomycins	*fosA3*	10.32	0.00	0.00	37.50	0.00	41.18	0.00	22.22	0.00
	*fosA7*	0.00	0.00	0.00	0.00	0.00	0.00	0.00	0.00	14.52
Lincosamides	*lnu(F)*	0.00	0.00	4.44	25.00	0.00	70.59	0.00	66.67	8.06
Polymyxins	*mcr-1.1*	0.00	0.00	0.00	50.00	0.00	0.00	0.00	11.11	0.00
Macrolides	*mph(A)*	0.00	8.70	4.44	75.00	0.00	41.18	40.00	22.22	6.45
	*mph(E)*	0.00	4.35	0.00	12.50	0.00	0.00	0.00	0.00	1.61
	*msr(E)*	0.00	4.35	0.00	12.50	0.00	0.00	0.00	0.00	1.61
Quinolones	*oqxA*	0.00	4.35	0.00	37.50	0.00	0.00	6.67	0.00	1.61
	*oqxB*	0.00	4.35	0.00	37.50	0.00	0.00	6.67	0.00	1.61
	*qnrB19*	0.00	0.00	0.00	0.00	0.00	0.00	0.00	0.00	12.90
	*qnrB6*	0.00	0.00	2.22	0.00	0.00	0.00	33.33	0.00	3.23
	*qnrS1*	7.14	34.78	26.67	0.00	0.00	23.53	40.00	77.78	29.03
	*qnrS2*	0.00	4.35	2.22	12.50	0.00	0.00	0.00	0.00	1.61
	*aac(6′)-Ib-cr*	0.00	4.35	13.33	75.00	0.00	0.00	40.00	0.00	3.23
Tetracyclines	*tet(A)*	26.19	43.48	13.33	87.50	33.33	88.24	73.33	44.44	32.26
	*tet(B)*	0.00	0.00	80.00	0.00	0.00	0.00	0.00	0.00	1.61
	*tet(M)*	0.00	30.43	0.00	0.00	0.00	0.00	0.00	0.00	8.06

**Table A2:** Occurrence of quinolone target gene mutations in predominant *Salmonella* serovars.

Gene	Quinolone Target Gene Mutation Rate (%)								
	*S.* Enteritidis (*n* = 126)	*S.* Typhimurium (*n* = 23)	*S.* 4,[5],12:i:- (*n* = 45)	*S.* Indiana (*n* = 8)	*S.* Infantis (*n* = 9)	*S.* Kentucky (*n* = 17)	*S.* London (*n* = 15)	*S.* Thompson (*n* = 9)	Others (*n* = 62)
*gyrA*	94.44	52.17	4.44	100	33.33	100	13.33	11.11	11.3
*gyrB*	0	0	0	0	0	0	0	0	0
*parC*	5.56	0	4.44	87.5	100	94.12	73.33	88.89	88.71
*parE*	0	0	0	0	0	0	0	0	0

**Table A3:** Mean number of genetic elements in different *Salmonella* serovars.

Index	*S.* Enteritidis (*n* = 126)	*S.* Typhimurium (*n* = 23)	*S.* 4,[5],12:i:- (*n* = 45)	*S.* Indiana (*n* = 8)	*S.* Infantis (*n* = 9)	*S.* Kentucky (*n* = 17)	*S.* London (*n* = 15)	*S.* Thompson (*n* = 9)	Others (*n* = 62)
Mean number of ARGs	4.30 ^a^	5.70 ^ab^	7.73 ^ab^	15.75 ^c^	4.00 ^a^	11.94 ^b^	9.20 ^b^	11.67 ^b^	5.44 ^a^
Mean number of IS elements	16.91 ^a^	17.83 ^a^	18.13 ^a^	22.25 ^b^	19.22 ^a^	20.47 ^b^	22.33 ^b^	20.44 ^b^	18.18 ^a^
Mean number of VFs	111.17 ^c^	112.13 ^c^	107.06 ^bc^	98.13 ^a^	108.11 ^b^	103.97 ^ab^	105.33 ^ab^	103.89 ^ab^	100.77 ^a^

Footnote: Different superscript letters (a, b, c) in the same row denote statistically significant differences among serovars based on Dunn’s test after the Kruskal–Wallis test (*p* < 0.05). Serovars sharing the same letter are not significantly different.

**Table A4:** Distribution of *Salmonella* Serovars by Source: Food and Stool Cases.

Salmonella Serovar	Number of Isolates from Food Sources	Number of Isolates from Stool Samples	Total Number of Isolates
*S.* Enteritidis	73	53	126
*S.* 4,[5],12:i:-	7	38	45
*S.* Typhimurium	14	9	23
*S.* Kentucky	16	1	17
*S.* London	7	8	15
*S.* Infantis	7	2	9
*S.* Thompson	7	2	9
*S.* Indiana	8	0	8
